# Illness perceptions and their relation to physical activity in children and adolescents with hemophilia

**DOI:** 10.1080/21642850.2020.1823226

**Published:** 2020-10-04

**Authors:** Sarah Bérubé, Claudine Amesse, Serge Sultan

**Affiliations:** a Université de Montréal, Montreal, Canada; b Sainte-Justine University Health Centre, Montreal, Canada

**Keywords:** hemophilia, illness perceptions, physical activity, common-sense model, self-regulation, children and adolescents

## Abstract

**Background:** Hemophilia is a rare bleeding disorder in which illness perceptions have seldom been studied. Illness perceptions are important in predicting patients’ behavior. Due to the risk of bleeding and joint damage, healthcare professionals often discourage some physical activities. Those restrictions can be difficult to follow for pediatric patients. This study investigates the relationship between illness perceptions, physical activity behavior, and intentions amongst this population.

**Methods:** Twenty-four 6–18-year-old patients with severe hemophilia completed the Brief Illness Perception Questionnaire. A questionnaire assessing their level of physical activity and intentions toward safe and higher-risk physical activity was also administered. Clinical and socio-demographic data were collected. Associations were studied using hierarchical clustering of physical activity patterns, and Mann–Whitney U comparisons between clusters.

**Results:** Perceptions ranged from slightly to moderately threatening, and 20–30% of participants had highly threatening perceptions on Consequences, Identity, Concern, and Emotional response. The subgroup who engaged in more high-risk physical activity and had stronger intentions to engage in this type of activity also held more concerns and perceived more symptoms.

**Conclusion:** Patients at risk of non-adherence to recommendations concerning physical activity have more threatening illness beliefs that could be addressed during specific interventions or routine appointments. Addressing illness beliefs could be an element to behavior change. Strong emotional responses to hemophilia also stresses the need for psychosocial support strategies.

## Introduction

Hemophilia is a rare bleeding disorder in which the blood does not clot normally. Hemophilia A and B are the two most common forms and involve coagulation factors VIII and IX respectively. In severe hemophilia, the coagulation factor’s concentration in the blood is less than 1%, which exposes these patients to a higher risk of internal bleeding, especially in the joints (Srivastava et al., 2013). Bleeding episodes in the joints, if repeated, can lead to important and permanent joint damage (Gringeri, Ewenstein, & Reininger, 2014). Although hemophilia is not curable, bleeding episodes can be prevented by appropriate self-care. Infusions of the missing factor protein on a regular basis (prophylaxis) or when needed (on demand) are usually at the core of the treatment plan (Srivastava et al., 2013). In addition to replacement therapy, the official guidelines of the World Federation of Hemophilia also encourage the regular practice of safe physical activity (PA) (Srivastava et al., 2013). Being physically active helps prevent joint bleeds by strengthening the muscles around the joints, amongst other benefits. However, certain types of PA are strongly discouraged by healthcare professionals and they vary depending on the severity of the hemophilia, the level of joint health, and the strength and coordination of the patient. Sports involving contact and speed commonly fall into this category because they might cause bleeding in the joints (Cailly Howell & Patel, 2017; Zourikian, Jarock, & Mulder, 2010). Throughout this manuscript, PA that poses a higher risk of bleeding for a specific patient will be referred to as a high-risk activity, as determined by healthcare professionals.

Limitations in sports are a common concern for children and adolescents with hemophilia (Limperg et al., 2016). The practice of higher-risk activities is common in pediatric hemophilia (Ross, Goldenberg, Hund, & Manco-Johnson, 2009). Social pressures and the desire to be perceived as ‘normal’ can lead patients to engage in sports that are discouraged by their healthcare team (Bérubé, Cloutier-Bergeron, Amesse, & Sultan, 2017; Williams & Chapman, 2011). Indeed, children and adolescents with hemophilia usually practice more moderate- to higher-risk activities as compared to adults living with the same condition (Forsyth et al., 2014). Another issue arises when young patients avoid practicing PA of any kind, which also exposes them to a higher risk of joint bleeds (Nazzaro, Owens, Hoots, & Larson, 2006). Preventing joint bleeding is important as only two bleeding episodes in the same joint could cause permanent damage, such as a limitation in the range-of-motion of a joint and chronic pain. Moreover, bleeding episodes have a negative impact on the quality of life of pediatric patients (Bullinger & von Mackensen, 2003; A. v. Gringeri et al., 2004; Poon, Doctor, & Nichol, 2014; Shapiro et al., 2001).

According to the Common Sense Model of Self-regulation (CSM), the way patients view, understand, and feel about their illness (illness perceptions) are important factors in determining their motivation to follow their treatment plan (Leventhal, Meyer, & Nerenz, 1980). These factors will influence the way they cope with their condition and which actions they may take. Illness perceptions have long been studied in health psychology and have been shown to predict adherence, coping, and health outcomes in adult and pediatric patients (Broadbent et al., 2015; Hagger & Orbell, 2003; Law, Tolgyesi, & Howard, 2014). Some children may have difficulty in realistically conceiving the illness and understanding why it is necessary to follow recommendations of their healthcare team due to their level of cognitive development (Bir & Podmore, 1990). In fact, children and adolescents have been found to be more focused on current symptoms, short-term treatment gains, and more immediate consequences than adults (Law et al., 2014).

Illness perceptions are made of different components: identity beliefs (the extent to which the patient attributes symptoms to the illness), causal beliefs (the perceived cause of illness), timeline beliefs (the perception of the likely duration of the illness), beliefs about personal control and treatment control (whether the illness can be cured or controlled by one’s action and by treatment), consequences (perceived consequences on one’s life), understanding (the level of understanding of the illness), and emotional representations (emotional reactions to the health threat such as concerns, fear, anger, and distress) (Broadbent, Petrie, Main, & Weinman, 2006; Cameron & Leventhal, 2003). The relation between illness perceptions and self-management have been investigated in several studies in adults as well as in a pediatric patients (Broadbent, Donkin, & Stroh, 2011; Law et al., 2014). According to the model, the perception of a health threat will activate self-regulation processes (Cameron & Leventhal, 2003). Concordantly, some studies have found that stronger illness perceptions were associated with better adherence (Ross, Walker, & MacLeod, 2004; Van Os, Troop, Ryder, & Hart, 2018). However, according to the theory, when negative emotion arousal is too high or the health threat is perceived as unmanageable, affect regulation could become primary and lead to defensive denial vs to the management of the objective threat (e.g. through adherence) (Cameron & Leventhal, 2003). In the literature, more threatening beliefs are often linked to poorer health outcomes, poorer adherence to a health regimen, and lack of self-care behaviors, including PA in adults, children and adolescents (Broadbent et al., 2011; Broadbent et al., 2015; Law et al., 2014; Mosleh & Almalik, 2016; Skinner et al., 2003). Studies that focused on exercise found that more exercise was associated with a higher perception of control as well as a better understanding of the disease (Broadbent et al., 2011; Mosleh & Almalik, 2016; Skinner et al., 2003). In hemophilia studies, having less concern was associated with better adherence to treatment in adolescent and adult patients (Lamiani et al., 2015; Van Os et al., 2018). Some contradictions were found: having a stronger emotional reaction (e.g. fear and anger) and a stronger perception of chronicity were associated with both a better and a worse adherence to treatment in two studies (Lamiani et al., 2015; Van Os et al., 2018). None of those two studies focused exclusively on pediatric patients. The contradiction in the direction of the association could be explained by the use of different versions of the questionnaire, and differences in the level of perceived emotions and chronicity, as well as in health status, age, and treatment regimen (Lamiani et al., 2015; Van Os et al., 2018).

Illness perceptions may play a key role in motivation for safe practice of PA in children and adolescents with hemophilia. Paying a closer look at them would allow us to better understand the experience of pediatric patients and, consequently, help practitioners develop targeted interventions to modify perceptions and influence behaviors. Very few studies either directly investigate younger patients’ point of view on their hemophilia or focus on PA’s psychosocial determinants. To our knowledge, no study has used illness perceptions to understand PA with this population. This is all the more important as interventions aiming at changing illness perceptions have led to significant outcomes in patient health and wellbeing, and have been shown efficient in changing self-care behaviors such as exercise (Broadbent et al., 2015; Jones, Smith, & Llewellyn, 2016; Keogh et al., 2011; Petrie & Broadbent, 2003; Petrie, Cameron, Ellis, Buick, & Weinman, 2002).

In this study, we first wanted to describe illness perceptions in children and adolescents with hemophilia, and, second, to examine the relationship between illness perceptions and usual practice of PA, and between illness perceptions and intentions to practice PA in the future (since it has been shown to be a proximal predictor of actual behavior) (Armitage & Conner, 2001). In order to do so, we wanted to determine if there were consistent profiles in our sample with respect to how children behave and think about recommended and high-risk PA, and investigate whether illness perceptions would differ across these profiles. Based on studies on the relationship between PA and illness perceptions in other illnesses, as well as studies on other self-care behaviors, we expected that illness perceptions in general would differ across profiles of PA, i.e. that more negative illness perceptions would be associated with poorer adherence to PA recommendations.

## Materials and methods

### Participants

Patients aged 6–18 years with severe hemophilia A or B (VIII / IX < 1%), treated at Sainte-Justine University Health Center and accompanied by one of their parents, were approached for this study which consisted of a one-time assessment during their appointment at the hemophilia clinic. Contacting all of the eligible patients respected the natural variation in participants’ age, hemophilia type, treatment plan, etc. All of them spoke French. Patients with a diagnosis of less than a year or with the presence of a severe psychiatric disorder were excluded from this study. Questions were read to patients and a research assistant collected the answers. Young children were asked if they understood the questions before answering and all participants were encouraged to ask questions at any time during data collection. Children were assured that their answers would be kept confidential and they all, as well as their parents, gave their written informed consent before the study. The Sainte-Justine UHC Research Ethics Board approved this project.

### Measures

To assess illness perceptions, we used the Brief-Illness Perception Questionnaire (Brief-IPQ), a short version of the Illness Perception Questionnaire-Revised (Broadbent, Petrie, et al., 2006; Moss-Morris et al., 2002). This questionnaire contains eight questions, each rated on a 11-point Likert scale assessing illness perceptions of the CSM, as described in the introduction section: Consequences, Timeline, Personal control, Treatment control, Identity, Concern, Understanding, and Emotional response. The last and open-ended question about the believed causes of the illness was not included, as hemophilia is a genetic condition generally diagnosed early in life. The Brief-IPQ is a measure with highly adequate psychometric properties. A meta-analysis concluded that this instrument is suitable for a range of conditions and has good concurrent and predictive validity (Broadbent et al., 2015).

To assess behaviors regarding PA, we first used an open-ended question inquiring which sports had been specifically recommended and discouraged to patients by their healthcare team. Then, participants had to report their PA level in a typical week for both types of activities (safe and high-risk). In this article, high-risk PA is defined as any physical activity that has been identified as posing a high risk of bleeding for the patient by healthcare professionals and therefore should be avoided according to one’s treatment plan. These activities might differ between participants. Furthermore, considering that activities vary greatly according to the Canadian seasons, we inquired for both winter and summer activities and averaged both sets of results. We used the similar wording used in the Godin Leisure-Time questionnaire (Godin & Shephard, 1985): *In a typical week, how many days per week do you engage in this type of activities for more than 15 min?* Since the estimation of PA is not reliable for children under 10 years old, parents’ estimations were used for these participants (Kohl, Fulton, & Caspersen, 2000).

Intentions to adopt future behaviors were also assessed as a measure of patients’ motivation to follow recommendations in the future. We used one item for each type of PA (recommended or non-recommended). Following standard practice, the participant responded on a 7-point Likert scale, for example: *In the future, I intend to practice recommended/discouraged PA, 1-strongly disagree to 7-strongly agree* (Ajzen, 2006).

### Statistical analysis

For the scoring of the Brief-IPQ and descriptive statistics, we reversed scores for items 3, 4, and 7 as indicated in the scoring instructions (Broadbent, 2006).

In order to describe participants’ illness perceptions, we compared the mean score of our sample to the midpoint of 5 on the 11-point scale with a Wilcoxon signed-rank test to check whether responses were in the lower or upper half of the possible scale responses, representing respectively a lower perceived health threat (a more benign illness) or a higher perceived health threat (Broadbent, 2006). To explore possible profiles of behaviors and intentions toward PA in our sample, we used hierarchical clustering (Ward, squared Euclidean distance) (Beaulieu-Prévost, Ouellette, & Achille, 2005). This produced agglomerative clusters of participants who were similar on the selected internal variables. The internal variables used for clustering were the average number of days of safe and high-risk PA per week as well as intentions to practice safe and high-risk PA in the future. The final number of clusters was determined by the study of the dendrogram and through the verification of the clusters’ clinical interpretation (Mooi & Sarstedt, 2011). We compared illness perceptions across PA clusters using Mann–Whitney U tests. Effect sizes of the differences (*r)* were also calculated and interpreted according to Cohen’s convention of small (*r *= 0.1), medium (*r *= 0.3), and large (*r *= 0.5) effect sizes (Cohen, 1992).

## Results

All 31 patients who were on the list of the local hemostasis center and those who met the inclusion and exclusion criteria were approached to participate in this study. Of those, 26 patients (84%) and their parents accepted to participate (Table 1). Reasons for participation refusals were due to not having a scheduled appointment at the clinic (*N *= 3) and lack of time (*N *= 2). Two participants were excluded from our study because of comorbidities (intellectual disability and severe attention deficit disorder), resulting in a final sample size of 24 pediatric patients with a mean age of 11.8 ± 3.3 years. All participants were on prophylaxis or immune tolerance.

**Table 1.** Sample description (*n *= 24).

Characteristics	Mean (*SD*), Range	*n* (%)
Age (years)	11.8 (3.3), 6–18	
Country of birth		
Canada		20 (83%)
Other		4 (17%)
Type of hemophilia		
A (severe)		21 (88%)
B (severe)		3 (13%)
Type of treatment		
Prophylaxis		20 (83%)
Immune tolerance		4 (17%)
Bleeding episodes (past year)	4.3 (6.3), 0–24^a^	

^a^In our sample, 4 participants did not experience any bleeding in the last year.

**Figure 1.**
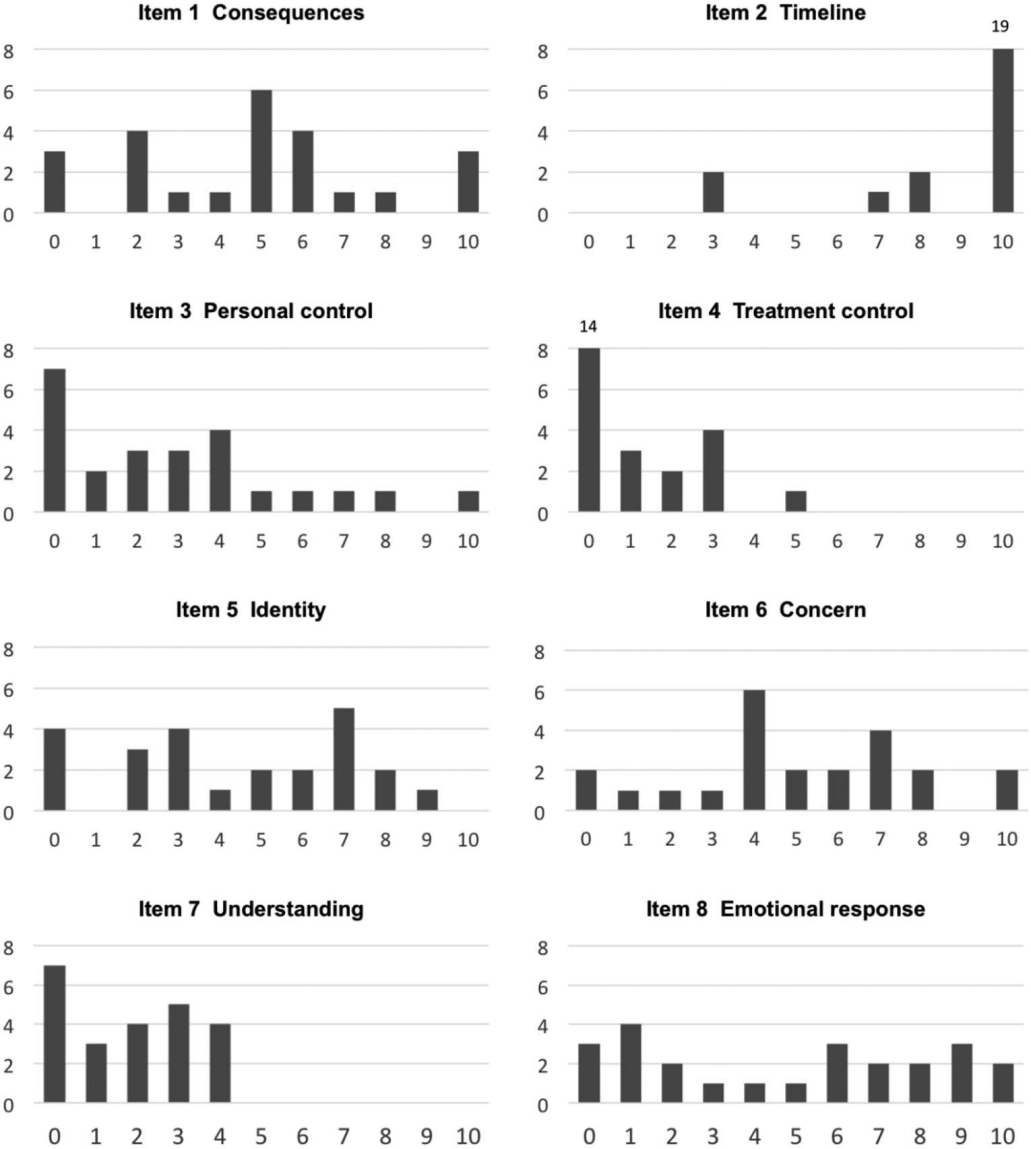
Frequency of participants’ answers (vertical axis) for each scale value (horizontal axis) on items of the Brief Illness Perception Questionnaire (Brief-IPQ).

Table 2 shows participants’ mean scores for each of the illness perceptions of the Brief-IPQ (for median scores and interquartile ranges, see supplementary file 2). Low scores represent rather positive beliefs and high scores show the more threatening ones. Participants agreed that the illness was chronic (high mean for Timeline and small SD). According to the distribution of responses in Figure 1, two participants did not perceive their illness as being chronic. Participants’ scores for Treatment control and Understanding were all equal or inferior to the midpoint of 5, thus in the portion of the scale representing a lower perceived threat. Mean score for Personal control was also under the midpoint of 5, although a few participants had higher scores (Wilcoxon, *p *< 0.01). This suggests that, in general, patients perceived having a relatively good control over their illness, believed that their treatment was helpful, and that they had a good understanding of their hemophilia. However, Consequences, Identity, Concern, and Emotional response were rated as representing a higher threat for participants. For these items, participants rated these perceptions as being moderately strong, i.e. they perceived a moderate level of consequence and symptoms, and experienced a moderate level of concern and other negative emotions in relation to their illness. There was a significant variability in answers to these aspects and about 20–35% of the sample answered 7 or more on the 0–10-point scale, with higher scores representing more threatening beliefs.

**Table 2.** Mean, standard deviation, and range on items of the Brief-IPQ for all participants (*N *= 24), Risk Profile (*n *= 11), and Safe Profile (*n *= 13).

Items	Description	Scale anchors	All	Risk Profile	Safe Profile	Effect size (*r*)
1	Consequences	none to severe	4.75(3.00), 0–10	5.45(3.33), 0–10	4.15(2.67), 0–10	0.24
2	Timeline	very short-term to forever	9.13(2.05), 3–10	8.91(2.21), 3–10	9.31(1.97), 3–10	0.14
3	Personal control^a^	excellent to no control	2.88(2.80), 0–10	4.00(2.97), 0–10	1.92(2.36), 0–8	0.38
4	Treatment control^a^	excellent to no control	1.00(1.44), 0–5	1.27(1.27), 0–3	0.77(1.59), 0–5	0.31
5	Identity	none to many severe symptoms	4.33(2.87), 0–9	5.82(1.99), 3–9	3.08(2.96), 0–8	0.47*
6	Concern	none to extreme	5.04(2.79)^b^, 0–10	6.70(2.36)^c^, 4–10	3.77(2.45), 0–7	0.51*
7	Understanding^a^	very clear to not at all	2.08(1.82), 0–5	2.64(1.86), 0–5	1.62(1.71), 0–5	0.28
8	Emotional response	none to extreme negative emotions	4.79(3.54), 0–10	6.18(3.28), 0–10	3.62(3.43), 0–9	0.36
Total	Threatening views (global score)		34.13(12.77)^b^, 4–55	41.80(9.82)^c^, 25–55	28.23(11.83), 4–46	0.52*

All items are rated on a 10-point Likert scale.

^a^Item score is reversed-coded, with higher scores representing more threatening illness beliefs.

^b^*n* = 23.

^c^*n* = 10.

Difference between groups (Mann Whitney U): *p < 0.05, **p < .01.

We conducted the hierarchical clustering procedure to explore individual profiles toward PA. Upon graphical examination of this dendrogram, we evaluated the greatest distance in which observations were combined (Mooi & Sarstedt, 2011). The most obvious solution when using this method was to use two clusters. These clusters were also clinically relevant since they represented two types of distinct attitudes toward high-risk PA: avoiding risk vs. engaging in it. Other clustering alternatives were not interpretable clinically and were thus discarded (see supplementary file 1). To characterize the two profiles, we compared participants’ levels on internal variables and found that they differed on their past practice of riskier PA and intentions to engage in these activities in the future (see Table 3). Patients in Profile 1 practiced more high-risk PA (2.6 vs. 0.6 days per week) and had stronger intentions to engage in high-risk PA in the future than Profile 2 (5.1 vs. 1.7). Consequently, Profile 1 was labeled as the Risk Profile and Profile 2 as the Safe Profile. The two profiles did not significantly differ both on typical practice of safe PA and on intentions to practice these types of activities in the future. Further analysis also showed that the two profiles did not significantly differ in age (12.0(3.2) years vs. 11.7(3.6) years) nor in the number of bleeding episodes in the last year (3.8(6.3) episodes vs. 4.7(6.7)).

**Table 3.** Characterization of physical activity (PA) profiles in a group of 24 young patients with hemophilia (mean and standard variation).

	Risk Profile	Safe Profile	Effect size (*r*)
Physical activity practice^a^			
Recommended	3.59 (1.87)	4.42 (1.98)	0.22
Discouraged	2.64 (2.34)	0.62 (0.79)	0.51*
Intention to practice PA			
Recommended	6.09 (0.83)	6.15 (1.68)	0.22
Discouraged	5.09 (1.45)	1.69 (1.49)	0.75**

^a^ Number of days of self-reported practice in a typical week (average score for winter and summer season).

Difference between groups (Mann Whitney U): *p < 0.05, **p < .01.

Note: We used hierarchical clustering (Ward, squared Euclidean distance). The internal variables entered for clustering were: average number of days of safe PA per week, average number of days of riskier PA per week, intention to practice safe PA in the future, and intention to practice riskier PA. This produced two agglomerative clusters of participants with similarities on the selected internal variables. Differences in mean scores for the two groups on the internal variables are presented in this table.

While exploring differences in illness perceptions across these profiles, we found that patients from the Risk Profile had significantly more threatening views of the illness overall (total score). They also reported more symptoms (stronger Identity), and were more concerned about their illness (stronger Concern) compared to their Safe Profile counterparts. Importantly, the size of these differences ranged from moderate to large (Cohen, 1992). We also performed further analysis to verify that the removal of the 4 participants that were on immune-tolerance therapy would not affect the present results. This was not the case with the exception of Concern, for which the difference did not reach significance (*p *= .53) when comparing the two profiles. It is unclear whether this difference in our results is due to individual differences, systematic differences between individuals with or without immune tolerance-therapy, or a reduction in statistical power.

## Discussion

In a study exploring illness perceptions and patterns of PA in 24 children and adolescents with hemophilia, we found that most dimensions of illness perceptions ranged from slightly to moderately threatening, with the exception of Timeline. Moderately strong perceptions were found in relation to Consequences, Identity, Concern, and Emotional responses. Moreover, there was a high variability in answers to these aspects and a significant proportion of participants (20–35%) estimated these negative perceptions as being very strong (7 or higher on the 11-point scale). On the other hand, participants were all rather positively confident regarding Treatment control and Understanding. Personal control was also high for most participants. We found consistent profiles describing behaviors and intentions toward PA in this sample, with the Risk Profile practicing more high-risk (discouraged by their healthcare professionals) PA and expressing stronger intentions to practice those activities in the future compared to the second group (Safe Profile). However, there was no significant difference in practice or intentions for safe PA. As for the associations between illness perceptions and individual profiles toward PA, participants in the Risk Group had significantly more threatening perceptions of their illness globally. They also perceived more symptoms of their illness and had more concerns related to hemophilia.

Our results highlight that, even though pediatric patients may be confident in the efficacy of their treatment and in their own capacity to control their condition, some aspects of hemophilia and its treatment can be particularly difficult for them on an emotional level. Physical dysfunctions, pain, the unpredictable nature of the illness, social challenges, and efforts to conceal their difference can all represent important sources of concern for pediatric patients with hemophilia (Nazzaro et al., 2006; Williams & Chapman, 2011). A large proportion of participants (35% or more) in our study expressed strong emotional representations of illness (7 or more on an 11-point scale), which is concerning considering that stronger emotional representations have been associated with depression, anxiety, and a worse quality of life in patients with diabetes, as reported in a meta-analysis on the Brief-IPQ (Broadbent et al., 2015). However, participants had a unanimously high level of Treatment control and Understanding, and a high level of Personal control, suggesting that healthcare providers and caregivers are sufficiently supportive in providing information about hemophilia and teaching children and adolescents with hemophilia about their medical treatment. Interestingly, these representations were similar between the Risk Profile and the Safe Profile. This could mean that youngsters who are inclined to engage in high-risk PA do not particularly feel the need to gain more information or to increase their self-care skills. Thus, they might not be interested in formal information-based interventions. Similarly, a meta-analysis on interventions for changing PA behaviors in children has concluded that education alone is unlikely to change behavior (Brown et al., 2016). However, some results also stress the need to objectively assess the degree of knowledge of patients, as some misconceptions can indeed be present and impede adherence to recommendations. The fact that a subsample in our study did not see hemophilia as a chronic illness was surprising. When exploring possible explanations for this phenomenon, we found a study on illness perceptions suggesting that adolescents and young adults with hemophilia have a tendency to see hemophilia as being less chronic than older adults do (Lamiani et al., 2015). However, younger age may not completely explain this misconception, as it may also be due to a lack of knowledge about the illness, which is independent of age. This explanation is supported by a study that has shown that patients of all ages, as well as caregivers, can lack basic and important knowledge about hemophilia and its treatment (Novais, Duclos, Varin, Lopez, & Chamouard, 2016). Furthermore, it would be important not to discard the possibility that these patients believed in an eventual cure considering the important advances in gene therapy (Herzog, 2016).

Despite the same average number of bleeding episodes in the last year in the two profiles, participants in the Risk Profile perceived more symptoms and were more concerned about their illness than those in the Safe Profile. Apart from the possibility that they experience more symptoms other than bleeding, e.g. chronic pain, a possible explanation is that children and adolescents in the Risk Profile were more vigilant concerning their hemophilia symptoms and were more concerned about their illness because they feared the negative consequences of their behaviors. This is consistent with the widespread approach of health professionals (including in our own center) that strongly emphasizes risks resulting from inappropriate PA. This would mean that freeing themselves from the constraints of their PA does not make them feel better in the long run. An alternative explanation lies in the use of denial by patients who perceive more symptoms. A meta-analysis that explored the relation between illness perception and coping behaviors found moderate to strong correlations between identity beliefs (perceiving more symptoms) and the use of avoidance/denial defined as the cognitive or behavioral attempts to ignore or avoid the existence of the problem or illness (Hagger & Orbell, 2003). This means that it is possible that children and adolescents with hemophilia who perceive many severe symptoms and are more concerned consciously or unconsciously choose not to adhere to recommendations in order to avoid the anxiety that comes with recognizing the presence of a chronic illness. Studies in other clinical populations have suggested that a moderate level of worry or fear arousal is optimal for a patient’s engagement in adequate health behaviors (Phillips, Green, & Morrissey, 2012; Strong & Dubas, 1993). In adults with hemophilia, not accepting one’s hemophilia has been recognized as a barrier to illness management, as self-care acts as a reminder of the illness (Schrijvers et al., 2015). There was also a trend for participants in the Risk Profile to perceive having less control over their illness and to experience more negative emotions. It is possible that the size of our sample was too small to reach statistical significance. Finally, the fact that there was no difference between the two profiles in terms of the number of bleeding episodes is somewhat counterintuitive, as one would expect that engaging in more high-risk PA would impact the number of bleeding episodes. A possible explanation resides in the total amount of PA. Participants in the Risk Profile tended to practice more PA overall when both recommended and high-risk activities were cumulated, benefits, such as stronger muscles around the joints,thus compensating for some risks of injury. It is also possible that participants’ behaviors had not been going on for long enough to yield an impact on joint bleeds in our sample.

If these results were to be confirmed in large-scale studies, they should be translated into recommendations for clinical practice. In fact, our study suggests that a subgroup of patients is particularly at risk of non-adherence to recommendations concerning PA and that this group has more threatening illness beliefs that could be addressed during specific interventions or routine appointments. According to this study, pediatric patients who engage in riskier PA experience more concern and perceive more symptoms regarding their illness. This is contrary to a misconception one could have that patients who do not follow the recommendations do not realize the risks involved in their behaviors. Therefore, a strategy to promote safer PA could be to assess the concerns and negative aspects they already experience rather than putting emphasis on communicating the risks associated with high-risk PA. This idea is in line with motivational interviewing techniques that use and amplify adolescents’ and adults’ own motivation for change in order to create behavioral change (Strait, McQuillin, Smith, & Englund, 2012). For children of all ages, acknowledging and being sensitive to their perspective and feelings is part of an autonomy-supportive approach, which has been shown to influence health behaviors (Bérubé, Mouillard, Amesse, & Sultan, 2016). Even though quality of life has been generally good in young patients with hemophilia since the introduction of home treatment, especially for adolescents on prophylaxis, a high percentage of our sample still reported having a strong emotional response to hemophilia, which stresses the need for psychosocial support strategies (A. Gringeri et al., 2004). Illness perceptions are rarely assessed by healthcare providers but individuals are generally open to discuss them (Petrie & Weinman, 2006). An interesting approach for discussing illness perceptions has been through peer group discussions, which allow children to express their inaccurate perceptions about the illness in order to then reframe them (Williams & Binnie, 2002). Other creative ways have been successfully used to assess illness perceptions such as the use of drawings (Broadbent, Ellis, Gamble, & Petrie, 2006).

It is important to recognize the limitations of this research. First, as hemophilia is a rare disease, our sample is limited and not randomly selected. Therefore, it is vulnerable to a lack of statistical power as well as selection bias, which may threaten external validity. Importantly, the study took place in Canada, where prophylaxis access is possible through the public healthcare insurance system. This decreases the financial burden and limits some of the stressors that might be present in families living with a child with hemophilia in other countries. Equally important, the correlational design of this study prevents us from drawing causal inferences between illness perceptions and PA patterns. Furthermore, our study included pediatric patients of a wide age range and our small sample size prevented us from studying differences between age groups. We recognized that patients from different ages have their own challenges often related to their developmental stage, and that the present study does not account for these specific differences. Also, PA practice and illness beliefs were not objectively assessed, and self-reports may have been influenced by social desirability. While the Brief-IPQ has been successfully used with children from the age of eight, it has not been validated for patients that young (Broadbent et al., 2015). Pediatric studies on antecedents of self-care behaviors are extremely rare, all the more so in hemophilia. Future studies should address these limitations by using longitudinal designs and more extensive measures of self-care including PA. Since PA patterns acquired in childhood and adolescence tend to persist into adulthood, understanding early PA determinants and its prevention are paramount (Hirvensalo & Lintunen, 2011; Tercyak, 2007).

To conclude, in a study of 24 pediatric patients with hemophilia, we surveyed illness perceptions, patterns of behaviors, and intentions toward PA. We found that children had slightly to moderately threatening illness beliefs and that a significant proportion had highly threatening beliefs. Furthermore, we found that children who practiced riskier PA and had the intention to do so in the future also felt more concerned about their illness and experienced their illness as more symptomatic than the rest of the sample. Future studies are needed to confirm these results and to investigate the interplay of motivation and behavior in this population.
